# Supplementary material for: Pneumococcal Surface Protein A-Hybrid Nanoparticles Protect Mice from Lethal Challenge after Mucosal Immunization Targeting the Lungs
Source: Pharmaceutics. 2022 Jun 11;14(6):1238. doi: 10.3390/pharmaceutics14061238 (PMC9230107; doi:10.3390/pharmaceutics14061238)
Supplement: Supplementary file 1 [file pharmaceutics-14-01238-s001.zip › pharmaceutics-1728424-supplementary.pdf]

Supplementary material

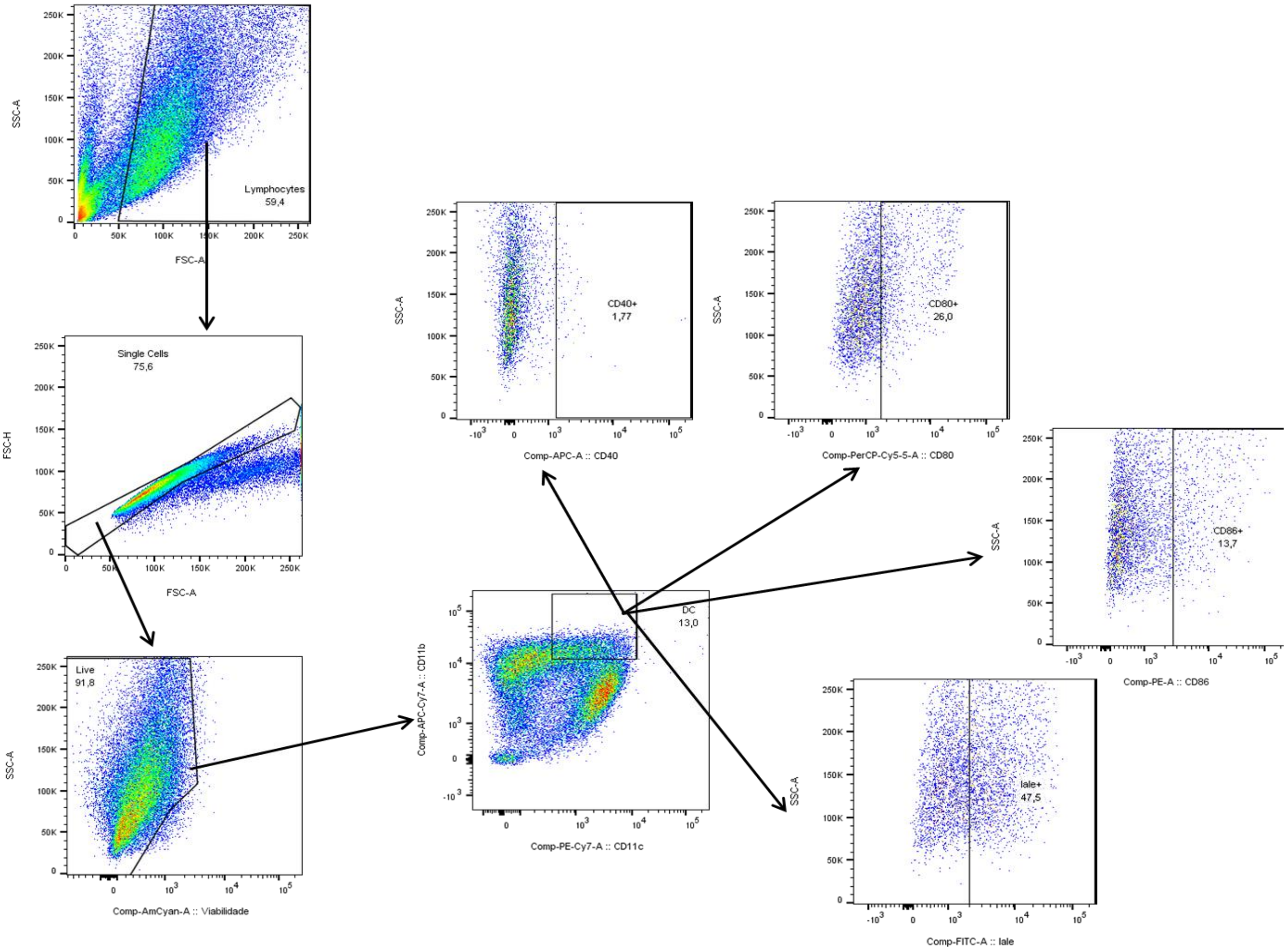

Figure S1. Gates applied to determine cell types by flow cytometry. First, lymphocytes were selected, then single and live cells. Dendritic cells (CD11b+/CD11c+) were labelled with CD11c PE-Cy7 and CD11b APC-Cy7 antibodies, CD40+ cells with CD40 APC, CD80+ cells with CD80 PerCP-Cy5.5, CD86+ cells with CD86 PE, and MHC Class II I-A/I-E+ cells with MHC Class II (I-A/I-E) FITC antibodies.

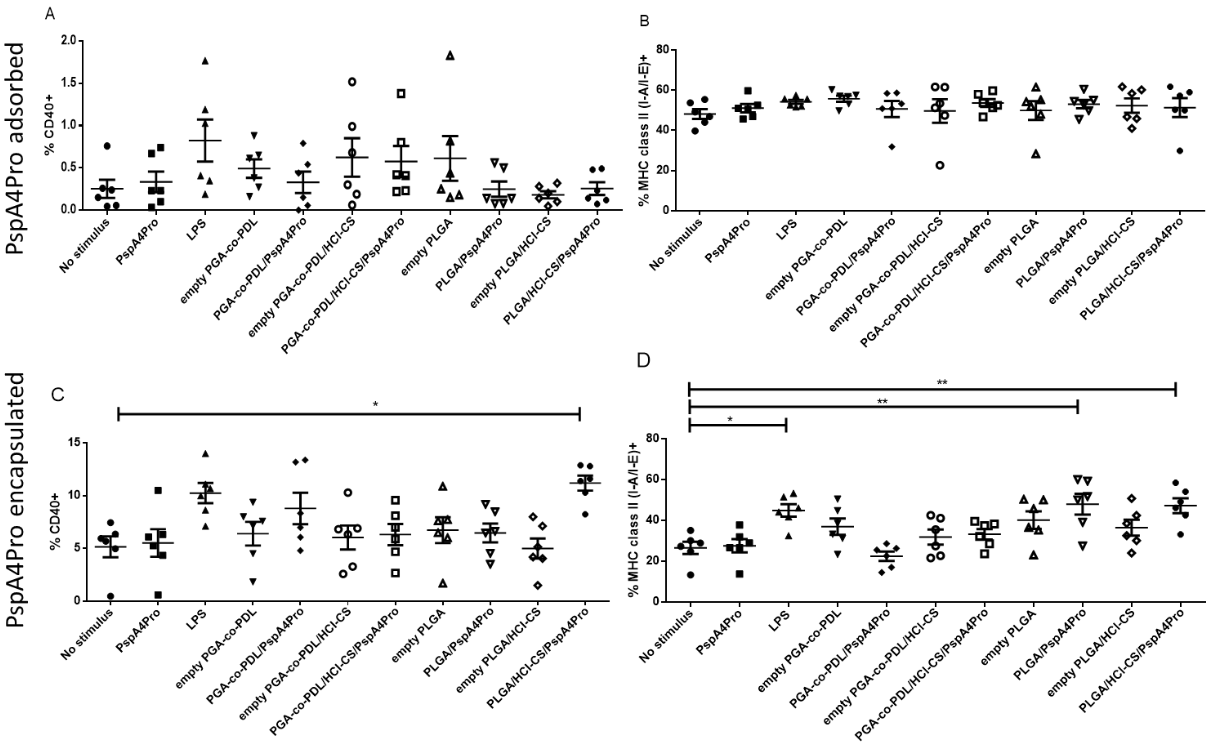

Figure S2. Activation of dendritic cells by NCMPs carrying different NP formulations containing adsorbed or encapsulated PspA4Pro**.** NPs were prepared with or without chitosan hydrochloride and contain PspA4Pro adsorbed onto the surface (A and B) or encapsulated into the NPs (C and D). Percentage of CD40+ DCs (A and C); percentage of MHC Class II I-A/I-E+ DCs (B and D). Significant differences in relation to the control group without stimulus are indicated (One-way ANOVA, Tukey's comparison test). *p<0.05, **p<0.01.
